# Supplementary material for: Real-world comparative study of drug retention of Janus kinase inhibitors in patients with rheumatoid arthritis
Source: PLoS One. 2024 Jul 11;19(7):e0306714. doi: 10.1371/journal.pone.0306714 (PMC11239012; doi:10.1371/journal.pone.0306714)
Supplement: S1 Fig — The starting point (0 years) is the date on which the observations began. DAS28-CRP: disease activity score 28 using C-reactive protein, JAKi: Janus kinase inhibitor; BARI: baricitinib; TOFA: tofacitinib; UPA: upadacitinib; No: number. (PPTX) [file pone.0306714.s001.pptx]

## Slide 1
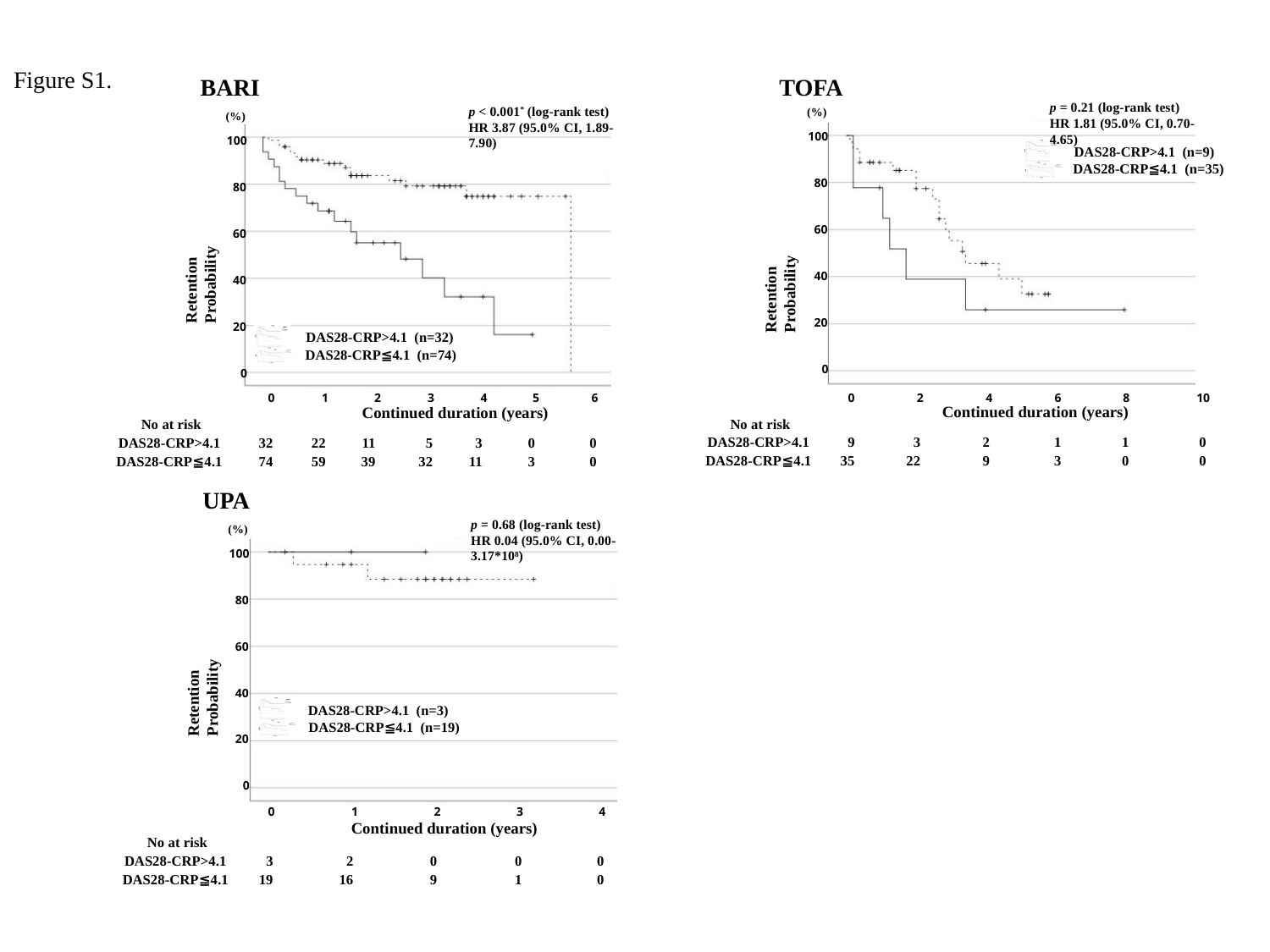

Figure S1.
TOFA
BARI
p = 0.21 (log-rank test)
HR 1.81 (95.0% CI, 0.70-4.65)
p < 0.001* (log-rank test)
HR 3.87 (95.0% CI, 1.89-7.90)
(%)
(%)
100
100
 DAS28-CRP>4.1 (n=9)
DAS28-CRP≦4.1 (n=35)
80
80
Retention Probability
Retention Probability
60
60
40
40
20
20
 DAS28-CRP>4.1 (n=32)
DAS28-CRP≦4.1 (n=74)
0
0
0
2
4
6
8
10
1
0
2
3
4
5
6
Continued duration (years)
Continued duration (years)
| No at risk | | | | | | |
| --- | --- | --- | --- | --- | --- | --- |
| DAS28-CRP>4.1 | 9 | 3 | 2 | 1 | 1 | 0 |
| DAS28-CRP≦4.1 | 35 | 22 | 9 | 3 | 0 | 0 |
| No at risk | | | | | | | |
| --- | --- | --- | --- | --- | --- | --- | --- |
| DAS28-CRP>4.1 | 32 | 22 | 11 | 5 | 3 | 0 | 0 |
| DAS28-CRP≦4.1 | 74 | 59 | 39 | 32 | 11 | 3 | 0 |
UPA
p = 0.68 (log-rank test)
HR 0.04 (95.0% CI, 0.00-3.17*108)
(%)
100
80
Retention Probability
60
40
 DAS28-CRP>4.1 (n=3)
DAS28-CRP≦4.1 (n=19)
20
0
0
1
2
3
4
Continued duration (years)
| No at risk | | | | | |
| --- | --- | --- | --- | --- | --- |
| DAS28-CRP>4.1 | 3 | 2 | 0 | 0 | 0 |
| DAS28-CRP≦4.1 | 19 | 16 | 9 | 1 | 0 |
