# Supplementary material for: Real-world comparative study of drug retention of Janus kinase inhibitors in patients with rheumatoid arthritis
Source: PLoS One. 2024 Jul 11;19(7):e0306714. doi: 10.1371/journal.pone.0306714 (PMC11239012; doi:10.1371/journal.pone.0306714)
Supplement: Attachment — Submitted filename: Response to reviewer2.docx [file pone.0306714.s003.docx]

Dear Reviewer #2,

Thank you for the time and effort that you have invested in reviewing the manuscript. We appreciate your suggestions and have revised the manuscript accordingly.

Reviewer #2: Dear authors

This manuscript shown that higher drug retention rates of JAK1 compared to those of pan-JAKis (baricitinib or tofacitinib) in real-world patients with RA. The discontinuation rates of pan-JAKi appeared to be higher in patients with high baseline RA disease activity. The authors honesty noted the limitations of this study include difference of observational period among JAKis, however, there are other concerns on this manuscript.

1. Regarding the multivariate analysis, the variables seem to be too many for the number of cases. This is a simple question, but would you clarify the reason for using those variables?

We appreciate your important comment.

We performed univariate and multivariate Cox regression analysis to identify factors associated with the AEs. Variables with p-values <0.2 in the univariate Cox regression analyses were entered in the multivariable Cox regression analysis. Discontinuations for all reasons occurred in 56 patients, and adverse event discontinuations occurred in 39 patients in this study.

Since the number of independent variables that can be analyzed in a multivariate Cox regression analysis is one per 10 events, analysis of up to 5-6 and 3-4 factors is appropriate for risk factors for discontinuation of medication for all reasons and for AEs, respectively. However, in this study, p<0.2 was included as a factor in the multivariate COX regression analysis, so five factors were entered into the analysis of risk factors for AE. As you indicated, we determined that too many factors were entered into the analysis of risk factors for AE. Explanatory variables must be narrowed down to an appropriate number prior to analysis. We chose to include factors that were p<0.2 in the univariate analysis in the multivariate Cox regression analysis for the analysis of risk factors of all discontinuations and AE. In cases where the number of variables that could be entered in the multivariate analysis was limited by the number of outcomes, the more significant variables were adopted.

We described the issue in the materials and methods of the revised manuscript, which can be found on lines 170-174, page 7.

“Variables with *p* < 0.2 were included in the multivariable Cox regression analysis for the analysis. In cases where the number of variables that could be entered in the multivariate analysis was limited by the number of outcomes, the more significant variables were adopted.”

In addition, we used age, gender, disease duration, rheumatoid factor (RF), anti-citrullinated protein antibody (ACPA), history of bDMARD use, coexisting diabetes mellitus (DM) or pulmonary disease, concomitant medications, and RA disease activity as factors in univariate Cox regression analysis. These factors 【age (Ref. PMID: 20039405, DOI: 10.1002/art.27227), gender (Ref. PMID: 26490106, DOI: 10.1093/rheumatology/kev374), disease duration (Ref. PMID: 30971306, DOI: 10.1186/s13075-019-1880-4), RF and ACPA (Ref. PMID: 26359449, DOI: 10.1136/annrheumdis-2015-207942), history of bDMARD use (Ref. PMID: 25922549, DOI: 10.1093/rheumatology/kev019), coexisting DM or pulmonary disease (Ref. PMID: 24414744, DOI: 10.1007/s00296-014-2945-y), concomitant medications (Ref. PMID: 20039405, DOI: 10.1002/art.27227) (Ref. PMID: 25922549, DOI: 10.1093/rheumatology/kev019), and RA disease activity (Ref. PMID: 25505001, DOI: 10.1093/rheumatology/keu455)】are already considered risk factors for discontinuation of biologics and JAKi.

We therefore used these variables in our analysis.

2. In all JAKis, prescribing at reduced dosages was noticeable, which was most particularly the case with baricitinib. This may also lead to a possible low overall continuation rate. Is there a possibility of significant results depending on the choice of explanatory variables?

We appreciate your important comment.

Kaplan-Meier analysis of the cumulative incidence of all discontinuations with and without reduced doses of all JAKi and baricitinib alone for rheumatoid arthritis patients showed no significant differences in either outcome (all JAKi; Log-rank p = 0.461, HR 0.82 (95%CI 0.48-1.40)/barcitinib alone; Log-rank p = 0.924, HR 0.97 (95%CI 0.47-1.97)).

In this study, we do not believe that the choice of explanatory variables for the reduced dosage of JAKi will yield significant results.

3. Despite the limitations of this paper, the retrospective and varying observation period, could you make additional statistical analysis of ROC analysis for drug discontinuation when the patiets have high disease activity at induction of JAKi and pan-JAKi use?

We appreciate your comment.

As you indicated, we performed ROC analysis of DAS28-CRP in all JAKi and pan-JAKi of all discontinuations. The cutoff value of DAS28-CRP for all JAKi discontinuations was 4.08 (sensitivity 43.6%, specificity 82.2%, area under the curve 0.61). Furthermore, the cutoff value of DAS28-CRP for all pan-JAKi discontinuations was 3.34 (sensitivity 62.3%, specificity 64.9%, area under the curve 0.61). The results suggest that high RA disease activity may be an important factor for total and adverse event discontinuation of JAKi.
